# Massive acute colonic pseudo-obstruction successfully managed with conservative therapy in a patient with cerebral palsy

**DOI:** 10.1186/1865-1380-4-15

**Published:** 2011-04-14

**Authors:** Derek R Cooney, Norma L Cooney

**Affiliations:** 1 Department of Emergency Medicine, SUNY Upstate Medical University, EMSTAT Center/550 East Genesee, Syracuse, New York 13202, USA

## Abstract

Acute colonic pseudo-obstruction (ACPO), also known as Ogilvie syndrome, is a massive dilation of the colon in the absence of mechanical obstruction. Treatment measures may include anticholinergic agents such as neostigmine, colonoscopy, or fluoroscopic decompression, surgical decompression, and partial or complete colectomy. We reviewed the case of a 26-year-old male with cerebral palsy who had a history of chronic intermittent constipation who presented to the emergency department (ED) with signs of impaction despite recurrent fleet enemas and oral polyethylene glycol 3350. The patient was found to have a massive colonic distention of 26 cm likely because of bowel dysmotility, consistent with ACPO. This article includes a discussion of the literature and images that represent clinical examination, x-ray, and computed tomography (CT) findings of this patient, who successfully underwent conservative management only. Emergency department detection of this condition is important, and early intervention may prevent surgical intervention and associated complications.

## Background

Acute colonic pseudo-obstruction (ACPO), also known as Ogilvie syndrome or acute colonic ileus, is a serious condition that can be relatively easily misdiagnosed and a patient's presentation ascribed to both minor conditions, such as functional constipation, and major conditions, like mechanical bowel obstruction. It is important for the emergency physician to be familiar with this entity and its management in order to avoid unnecessary morbidity in these cases.

Acute colonic pseudo-obstruction is a distention of the colon caused by decreased motility in the absence of mechanical obstruction. ACPO commonly occurs in association with a severe medical or surgical illness. Other causes include immobility, medications, electrolyte disturbances, and chronic illnesses that directly affect bowel motility. In an article by Vanek and Al-Salti, a review of 393 cases revealed a mean age in the mid to late 50s, and only 5.5% of patients presented without a known associated cause [1]. In this study 35.9% of the cases were associated with either a surgical or obstetrical procedure, and non-operative trauma was associated with 11.3% of cases. Untreated cases may result in the development of bowel perforation in up to 15%, resulting in a mortality rate of around 50% [2].

Cerebral palsy has been shown to be associated with a high rate of chronic constipation. An article by Veugelers et al. quotes an outpatient incidence as high as 74% in patients with CP, and there appears to be a neural component to the observed colonic dysmotility [3]. In a study by Johanson et al., neurological disease causing damage to the central nervous system was identified as an important independent risk factor [4]. These factors could predispose these patients to development of ACPO.

Symptoms of ACPO include nausea, vomiting, abdominal pain, constipation, diarrhea, and fever. Patients with the complications of ischemic bowel and perforation do not have significantly different presentations than those without them [1].

## Case presentation

**Figure 1:**
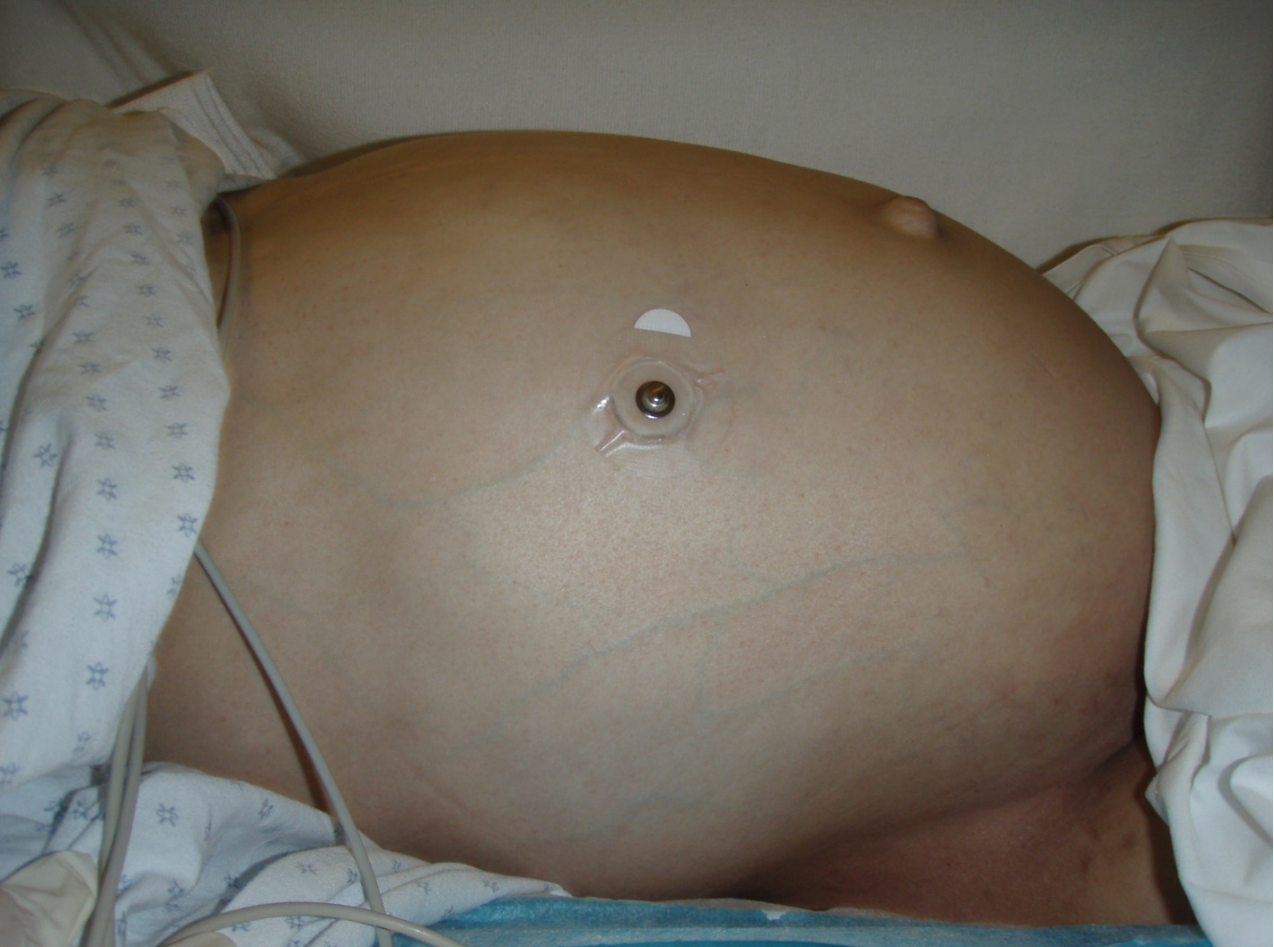
**Photo demonstrating severe abdominal distention**.

**Figure 2:**
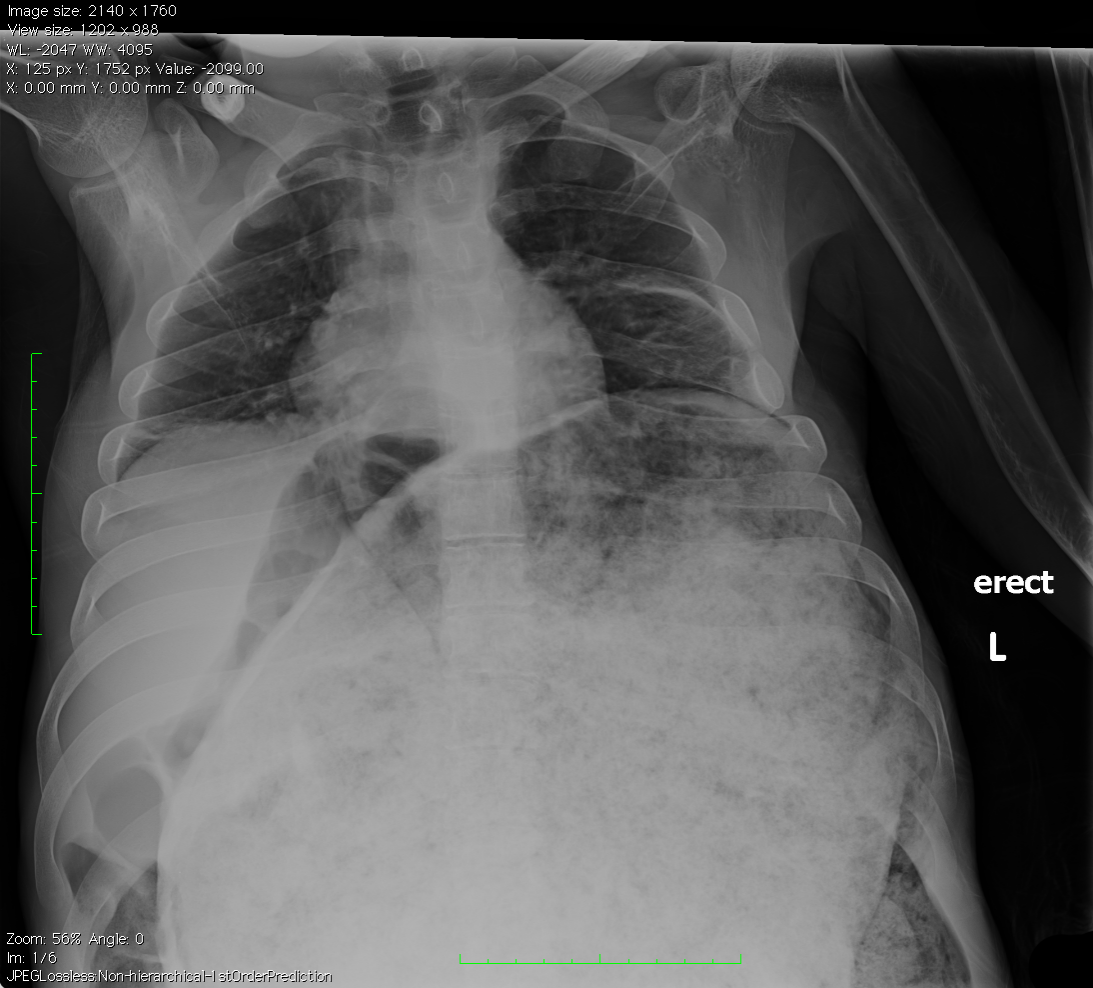
**X-ray revealing severe colonic dilatation from the pseudo-obstruction with large stool collection**.

**Figure 3:**
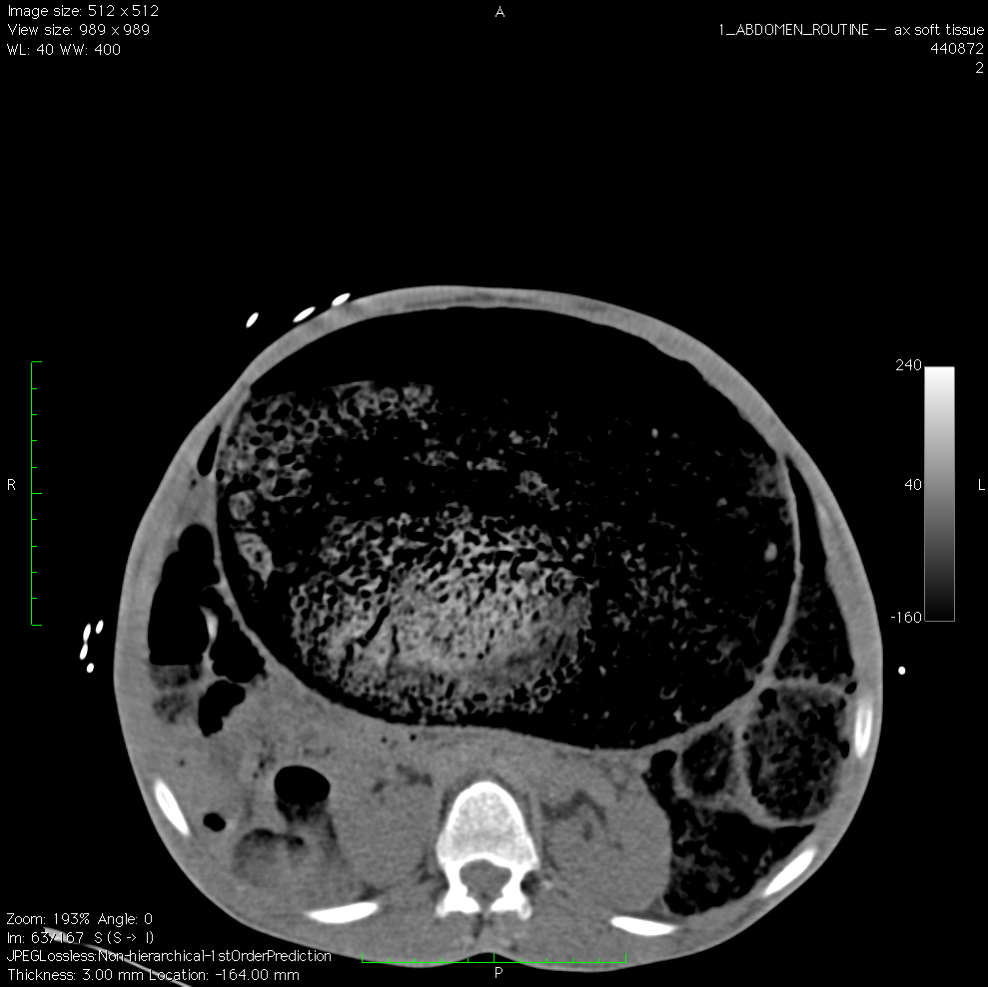
**Axial CT image of the pseudo-obstruction and severely dilated colon**.

**Figure 4:**
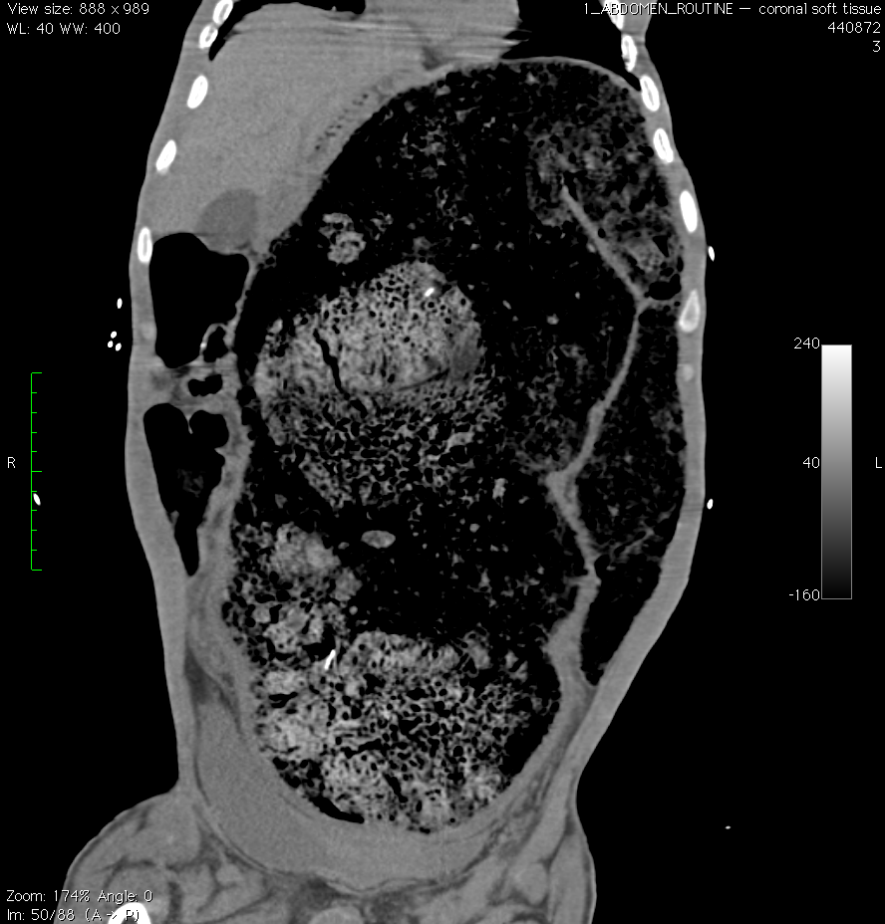
**Coronal CT image of pseudo-obstruction and severely dilated colon that almost completely fills the view of the abdomen cavity**.

**Figure 5:**
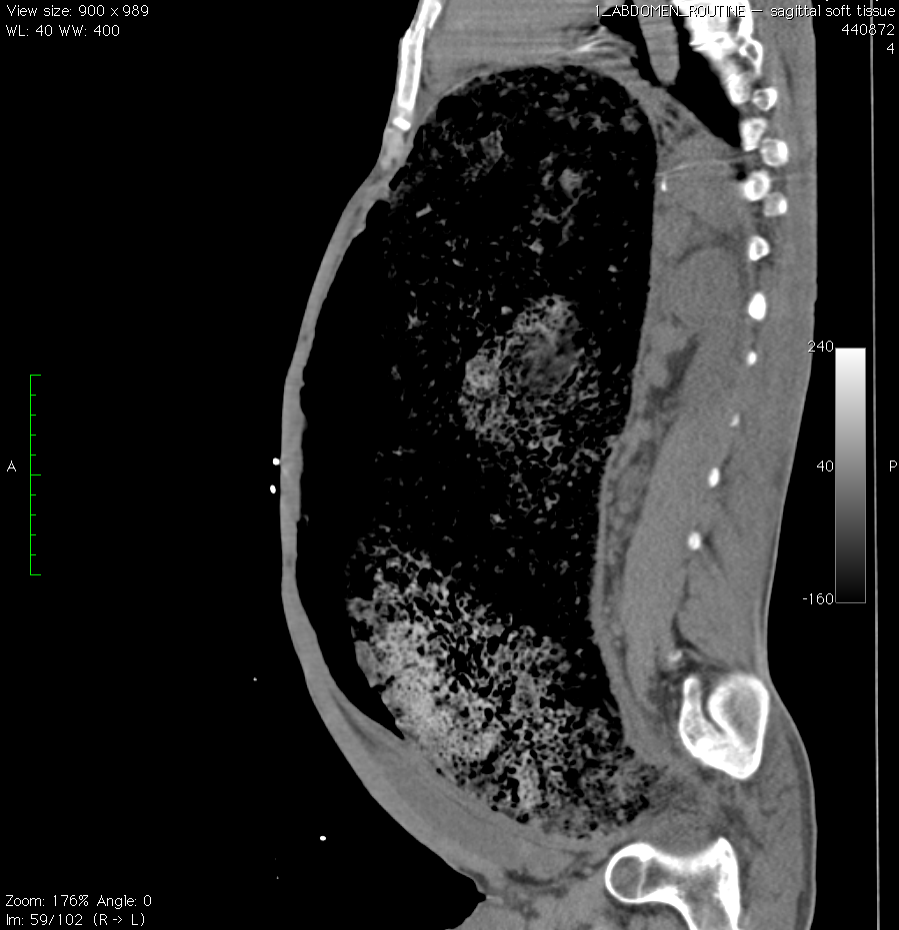
**Sagittal CT image of the pseudo-obstruction**.

A 26-year-old male with a history of cerebral palsy (CP) presented to the Emergency Department (ED) with the complaint of abdominal distension and constipation. The patient's mother was present and also the primary caregiver at home. The patient had a history of chronic intermittent constipation requiring weekly laxatives and fleets enemas. On this occasion, despite use of polyethylene glycol 3350 (an osmotic laxative), multiple enemas, and an attempt at manual fecal disimpaction by the mother, the patient had persistent constipation and discomfort. His vital signs were blood pressure: 148/85, heart rate: 150, respiratory rate: 20, and oxygenation saturation: 99% on room air, and he was afebrile. On exam, the patient had a decrease in mental status. His abdomen was markedly distended and rigid (Figure 1). Bowel sounds were absent. Laboratory studies showed no overwhelming abnormalities, with a white blood cell count of 13,000, creatinine level of 0.7, and potassium level of 3.7. An acute abdominal series showed a significantly distended colon with a 26-cm estimated diameter (Figure 2). CT of the abdomen showed a large amount of stool and air in the colon without evidence of a mechanical obstruction, bowel wall thickening, or signs of perforation (Figures 3, 4 and 5).

The patient was resuscitated in the ED with 2 l normal saline, and he was given intravenous antibiotics, piperacillin/tazobactam, to cover enteric bacteria for concern of impending bowel perforation and probable current microperforation. A nasogastric (NG) tube was placed. Gastroenterology and the general surgeon were immediately consulted. A gastrograffin enema was performed. No evidence of mechanical obstruction was visualized. As a precaution, the patient was admitted to the ICU for further management and care. The patient's white blood cell count rose to 26,000 on the second day. With NG tube decompression and multiple enemas, the patient eventually passed stool and gas. The colonic distention resolved without pharmacological, endoscopic, or surgical interventions. The patient did not develop worsening signs of sepsis or perforation, and was discharged in improved and stable condition.

## Discussion

The etiology of pseudo-obstruction is not clearly understood. However, it is known that the autonomic nervous system is the control center for bowel function. The parasympathetic system innervates the smooth muscle to induce peristalsis, thereby inducing normal defecation. Disruption of the parasympathetic system or innervation of the sympathetic system will disrupt normal bowel function. There are many conditions and interventions that have been shown to be associated with bowel dysmotility resulting in ACPO. The differential diagnoses for this condition should also include mechanical bowel obstruction, toxic megacolon, and severe constipation with fecal impaction. In the case described above, the patient's only significant risk factors for developing ACPO were his CP and overall chronic disability.

### Assessment

X-rays should be obtained immediately for a patient if one has concern about an obstructive process, especially if the suspicion is high for perforation. An acute abdominal series with an upright chest can provide vital information. Free air under the diaphragm indicating bowel perforation, differential air fluid levels indicating an ileus, and grossly dilated loops of bowel indicating an obstructive process can typically be seen and diagnosed from an x-ray. Additional information can be obtained from a CT regarding the location of the obstruction based on the transition zone.

Measurement of the colonic distention has been suggested as a potential guide to management and is routinely assessed radiographically. Studies have stated that dilation of the transverse colon of as little as 9 cm is potentially dangerous, and patients with cecal diameters >10-12 cm have been shown to be at higher risk of perforation [5-7]. In fact, the study by Vanek and Al-Salti reported no perforations for patients with <12 cm cecal diameter, a 7% perforation and ischemia rate for 12-14 cm, and 23% for patients with >14 cm cecal dilation [1]. However, some studies found no ischemia or perforation in patients with significant dilation beyond these limits [8]. Our own case reveals a 26-cm dilation of the colon without evidence of perforation or ischemia. Despite the massive dilation of the colon, the patient suffered no significant sequelae. Of additional interest, a retrospective study by Johnson et al. actually concluded that the duration of cecal distention may be associated with the perforation rate, but that the diameter was not. There may also be a significant difference in perforation risk in patients with severe colonic dilation with only moderate cecal dilation; however, no such comparison was found during a review of the literature.

### Management

The initial treatment for ACPO includes placement of a nasogastric tube, enemas, fluid resuscitation, and correction of electrolyte abnormalities. Antibiotics may be given to provide some coverage for patients who are suspected to have bowel ischemia or perforation [6]. However, in the study by Vanek and Al-Salti, there was almost no significant difference in symptoms at presentation between patients with these complications and those without [1], although they did note that patients with ischemia and/or perforation had a higher rate of fever (78%) than those that did not (31%). Conservative management is thought to be appropriate in patients without significant pain or dilation (<12 cm).

Anticholinergic agents such as neostigmine have been shown to have high success rates with restoration of peristalsis and have been used to treat ACPO successfully [9-11]. Trevisani showed clinical resolution of the acute pseudo-obstruction in 26 of 28 patients with the use of neostigmine [11]. Neostigmine enhances parasympathetic activity by competing with acetylcholine for attachment to acetylcholinesterase at sites of cholinergic transmission and enhancing cholinergic action. Side effects of neostigmine that may cause significant problems during acute management include increased abdominal pain, excess salivation, vomiting, bradycardia, asystole, hypotension, and seizures. In one article, 2 of the 11 patients treated with neostigmine required atropine for bradycardia [9]. Cardiac telemetry monitoring should be utilized during and after administration of this drug. The dose for neostigmine is 2 mg intravenously over 3-5 min with a cost of less than $10/dose. In a double-blinded placebo-controlled trial by Ponec et al., almost all (10 of 11) patients treated with neostigmine responded with the initial therapy, and none of the placebo group improved [9].

The need for colonoscopic decompression is routinely determined based on the severity of the pseudo-obstruction, and therefore early consultation with surgery or gastroenterology is appropriate. Colonic decompression may be indicated when the cecal diameter is >12 cm [12]. Despite this usual recommendation, our patient did well without this invasive procedure, and success rates may only be as high as 61-78% with a reported recurrence rate of 18-33% [6]. Iatrogenic perforation during this procedure has been reported as 3% [13]. Another invasive, non-surgical method for decompression is fluoroscopic-guided decompression [14].

Surgical interventions, such as tube cecostomy, cecostomy, ileostomy/colostomy, resection, exteriorization, intraoperative long colon tube, and exploratory laparotomy are reserved for patients who failed other management modalities. As expected, morbidity and mortality are greater in patients undergoing surgical interventions (30%/6%), when compared to those managed conservatively (14%/3%) or with colonoscopy (13%/2%) [1].

## Conclusion

Colonic pseudo-obstruction can be life-threatening, and if untreated may lead to perforation and a high rate of morbidity and mortality. Early consultation of a gastroenterologist and general surgeon is appropriate. Patients with large dilations may require pharmacologic, colonoscopic, fluoroscopic, or surgical intervention if conservative management fails. A review of the available literature and the results of this case seem to indicate that conservative management can be successful even in extreme cases. The responsibility of the ED physician is to make the appropriate diagnosis and initiate therapy to help decrease morbidity and mortality.

## Consent

Written informed consent was obtained from the patient for publication of this case report and accompanying images. A copy of the written consent is available for review by the Editor-in-Chief of this journal.

## Competing interests

The authors declare that they have no competing interests.

## Authors' contributions

NC participated in the care of the patient and provided case details, obtained consent, and obtained photographs. DC prepared images, reviewed reports and performed literature searches. Both DC and NC reviewed the literature and provided authorship of the text of this manuscript.
